# Crystal growth, structure elucidation and CHARDI/BVS investigations of β-KCoFe(PO_4_)_2_

**DOI:** 10.1107/S2056989022006521

**Published:** 2022-06-28

**Authors:** Adam Bouraima, Said Ouaatta, Jamal Khmiyas, Jean Jacques Anguilè, Thomas Makani, Abderrazzak Assani, Mohamed Saadi, Lahcen El Ammari

**Affiliations:** a Laboratoire de Chimie Appliquée des Matériaux, Centre des Sciences des Matériaux, Faculty of Science, Mohammed V University in Rabat, Avenue Ibn Batouta, BP 1014, Rabat, Morocco; b Laboratoire de Chimie des Matériaux Inorganiques, Faculté des Sciences, Département de Chimie, Université des Sciences et Techniques de Masuku, BP 943, Franceville, Gabon

**Keywords:** crystal structure, phosphate, zeolite-*ABW*, isotypism

## Abstract

Single crystals of β-KCoFe(PO_4_)_2_, potassium cobalt(II) iron(III) bis­(ortho­phosphate), were grown from the melt under atmospheric conditions. This phosphate crystallizes isotypically with KZnFe(PO_4_)_2_ in space group *C*2/*c*, adopting a zeolite-*ABW* type of structure. The structure of the present phosphate is distinguished by an occupational disorder of the two transition-metal sites with ratios Fe:Co of 0.5725:0.4275 for the first and 0.4275:0.5725 for the second site. In the crystal structure, PO_4_ and (Co,Fe)O_4_ tetra­hedra are linked through vertices to form elliptical rings with the sequence *DDDDUUUU* of up (*U*) and down (*D*) pointing vertices. Each eight-membered ring is surrounded by four other rings of the same type, delimiting inter­stices with rectangular shape. This arrangement leads to the formation of [(Co/Fe)(PO_4_)]^−^_∞_ sheets parallel to (001). Stacking of the sheets into a three-dimensional framework results in the formation of two types of channels. The first one is occupied by potassium cations, whereas the second one remains vacant. Calculations of bond-valence sums and charge distribution were used to confirm the structure model.

## Abstract 2

The crystal structure of β-KCoFe(PO_4_)_2_ is isotypic with that of KZnFe(PO_4_)_2_.

## 1. Chemical context

Transition-metal (*TM*) phosphates have been widely studied as potential candidates for various applications such as catal­ysis (Bautista *et al.*, 2007 ▸), ion exchange (Szirtes *et al.*, 2007 ▸), electrochemistry (Trad *et al.*, 2010 ▸) or as magnetic materials (Ofer *et al.*, 2012 ▸). In this context, zinc phosphates are of inter­est because the Zn^2+^ cation with its *d* ^10^ electronic configuration is susceptible to strong polarization and thus can be used to design new non-linear optical (NLO) materials (Shen *et al.*, 2016 ▸). In the family of transition-metal phosphate compounds, the anionic network is formed from PO_4_ tetra­hedra bonded to different types of coordination polyhedra of the form [*TM*O_*n*_] (*n* = 4, 5 and 6), leading to a wide variety of crystal structure types such as NaZnAl(PO_4_)_2_ (Yakubovich *et al.*, 2019 ▸). The structural diversity is mainly associated with the ability of *TM* cations to adopt different oxidation states with various types of coordination polyhedra (Moore & Ito, 1979 ▸; Hatert *et al.*, 2004 ▸).

It is in this context that our research team was involved with investigations of new phosphates with *A* ^I^, *M* ^II^ and *M* ^III^ cations where *A* is an alkali metal, and *M* ^II^ and *M* ^III^ are bivalent and trivalent cations, respectively. For example, Na_2_Co_2_Fe(PO_4_)_3_ (Bouraima *et al.*, 2015 ▸) and NaCuIn(PO_4_)_2_ (Benhsina *et al.*, 2020 ▸) are among the recently studied compounds. The present work is devoted to synthesis and crystal structure analysis of β-KCoFe(PO_4_)_2_, a new compound in the family of transition-metal phosphates.

## 2. Structural commentary

**Figure 1:**
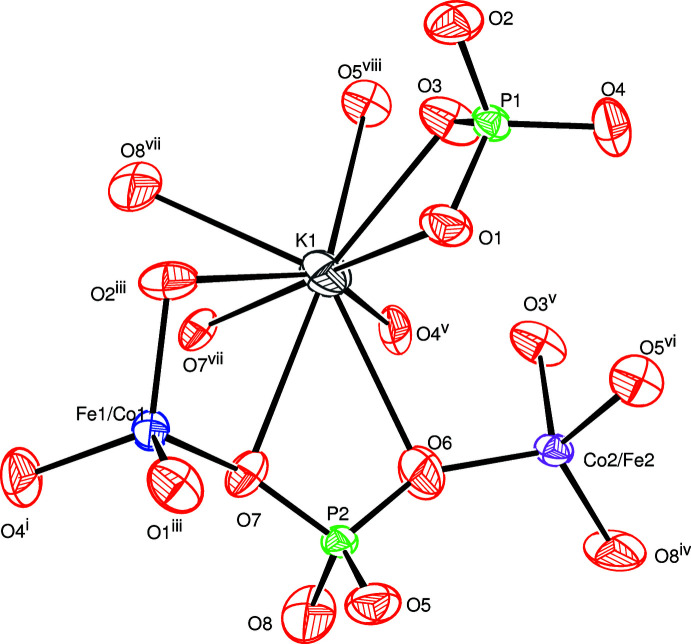
The principal building units in the crystal structure of β-KCoFe(PO_4_)_2_. Displacement ellipsoids are drawn at the 50% probability level. [Symmetry codes: (i) *x*, *y*, *z* + 1; (ii) *x*, −*y*, *z* + 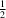 ; (iii) −*x* + 1, *y*, −*z* + 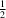 ; (iv) −*x*, *y*, −*z* + 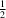 ; (v) −*x* + 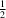 , −*y* + 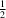 , −*z*; (vi) *x*, −*y*, *z* − 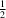 ; (vii) −*x* + 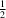 , −*y* + 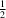 , −*z* + 1; (viii) −*x* + 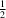 , *y* + 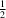 , −*z* + 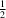 .]

The title compound crystallizes isotypically with KZnFe(PO_4_)_2_ (Badri *et al.*, 2015 ▸). The principal building units of β-KCoFe(PO_4_)_2_ are shown in Fig. 1 ▸, revealing that three types of more or less distorted tetra­hedra build up the framework structure. The two *TM* sites are characterized by partial disorder (see *Refinement*) with (Fe/Co)1—O distances varying between 1.877 (2) and 1.900 (2) Å and (Co/Fe)2—O distances between 1.881 (2) and 1.927 (2) Å. The two PO_4_ tetra­hedra are more regular with the P—O bonds lengths between 1.5172 (19) and 1.5306 (19) Å for P1O_4_ and 1.509 (2) and 1.533 (2) Å for P2O_4_.

**Figure 2:**
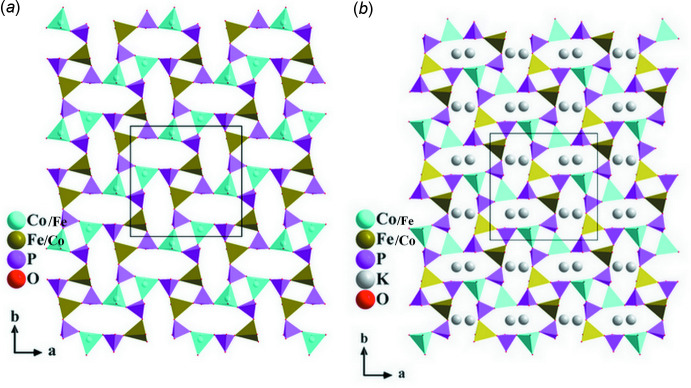
(*a*) A (001) layer at *z* ≃ 0 and (*b*) at *z* ≃ 0.5, resulting from vertex-sharing between *TM*O_4_ and PO_4_ tetra­hedra. Rings formed by eight corner-sharing tetra­hedra according to the sequence *DDDDUUUU* are shown on the left.

**Figure 3:**
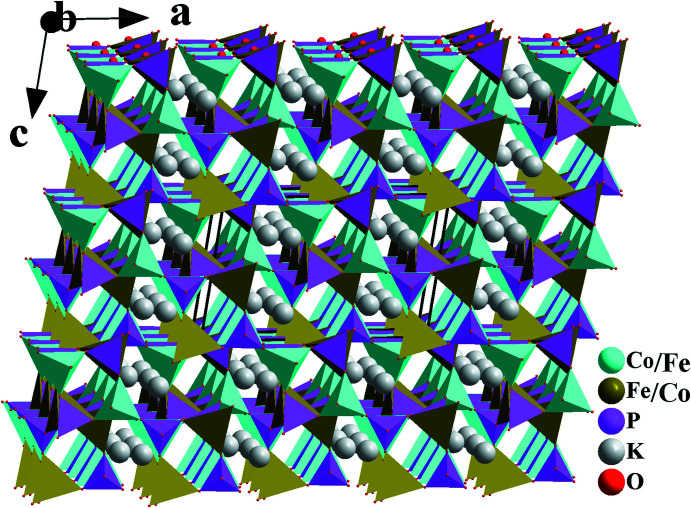
Perspective view of the crystal structure of β-KCoFe(PO_4_)_2_ approximately along [010], showing the channels in which the K^+^ cations are located.

The three different types of tetra­hedra are linked through vertices to form ellipse-shaped rings with the sequence *DDDDUUUU* of up (*U*) and down (*D*) pointing vertices, as shown in Fig. 2 ▸. Each eight-membered ring is surrounded by four other rings of the same type, delimiting two inter­stices with rectangular shape constituted by two PO_4_ and two (Fe/Co)1O_4_ tetra­hedra or two PO_4_ and two (Co/Fe)2O_4_ tetra­hedra. This assembly leads to the formation of [(Co/Fe)(PO_4_)]^−^_∞_ sheets extending parallel to (001) at *z* = 0, ½. Stacking of these sheets along [001] leads to the formation of a three-dimensional framework structure with two types of channels. The first one is occupied by potassium cations, whereas the second one remains vacant, as shown in Fig. 3 ▸. The K^+^ cation is surrounded by nine oxygen atoms with bond lengths between 2.694 (2) and 3.172 (2) Å.

Bond-valence sum (BVS) calculations (Brown, 1977 ▸,1978 ▸; Brown & Altermatt, 1985 ▸) and charge distribution (CHARDI) (Hoppe *et al.*, 1989 ▸) were used to confirm the structure model of β-KCoFe(PO_4_)_2_. BVS and CHARDI computations were carried out with *EXPO2014* (Altomare *et al.*, 2013 ▸) and *CHARDI2015* (Nespolo & Guillot, 2016 ▸), respectively. Table 1 ▸ compiles the valences *V*_(*i*)_ of cations determined with the BVS approach, as well as their corres­ponding charges *Q*_(*i*)_ calculated with the CHARDI concept. The data reveal that the values *Q*_(*i*)_ and *V*_(*i*)_ are all very close to the corresponding charges *q*_(*i*)_×sof_(*i*)_ (formal oxidation numbers *q*_(*i*)_ weighted by site occupation factors (sof_(*i*)_). For all cations, the inter­nal criterion *q*_(*i*)_/*Q*_(*i*)_ is very close to 1, and the mean absolute percentage deviation (MAPD) that evaluates the agreement between the *q*_(*i*)_ and *Q*_(*i*)_ charges is 0.3%, confirming the validity of the structural model (Eon & Nespolo, 2015 ▸). The global instability index (*GII*) was also used to check the plausibility of the crystal-structure model (Salinas-Sanchez *et al.*, 1992 ▸). The *GII* index evaluates the deviation of BVS parameters from the theoretical valence *V*_(*i*)_ averaged across all the constitutive atoms of the asymmetric unit. In an unstrained structure, *GII* is less than 0.1 and reaches 0.2 for those with lattice-induced deformations (Adams *et al.*, 2004 ▸). For the current crystal structure *GII* amounts to 0.1, indicating its stability.

## 3. Database survey

The phosphate KCoFe(PO_4_)_2_ crystallizes in two polymorphs in the same crystal system but with different unit-cell parameters and space groups. The *α*-form of KCoFe(PO_4_)_2_ reported by Badri *et al.* (2019 ▸) crystallizes in space group *P*2_1_/*c* with unit-cell parameters *a* = 5.148 (1), *b* = 14.403 (2), *c* = 9.256 (1) Å, β = 104.87 (2)°. The title compound crystallizes in space group *C*2/*c*. Whereas the environments around the two *TM* sites are tetra­hedral in the title compound, an octa­hedral coordination is found for one site (Co) in the *α*-form. The crystal structure of β-KCoFe(PO_4_)_2_ is isotypic with that of KZnFePO_4_)_2_ (Badri *et al.*, 2014 ▸), while that of *α*-KCoFe(PO_4_)_2_ is isotypic with those of KNiFe(PO_4_)_2_ and KMgFe(PO_4_)_2_ (Badri *et al.*, 2015 ▸).

## 4. Synthesis and crystallization

The phosphate β-KCoFe(PO_4_)_2_ was synthesized by mixing cobalt nitrate (Co(NO_3_)_2_·6H_2_O), iron nitrate [Fe(NO_3_)_3_·9H_2_O] ortho­phospho­ric acid (H_3_PO_4_) and potassium nitrate (KNO_3_) in molar ratios of 1:1:1:2. The mixture was placed in a small beaker containing distilled water and homogenized for 24 h. After evaporation to dryness, the reaction mixture underwent heat treatments at 573 and 773 K before being brought to fusion for crystal growth at 1223 K, followed by slow cooling. Crystals of purple color and of sufficient size for the analysis by X-ray diffraction were obtained from the final product.

**Figure 4:**
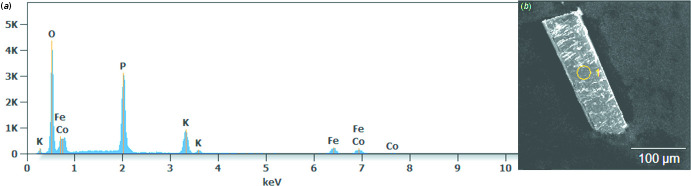
(*a*) EDS spectrum and (*b*) SEM micrographs of the title compound.

A Quattro ESEM scanning electron microscope (SEM) equipped with an energy dispersive X-ray spectrometer (EDS), operating under 20 kV accelerating voltage, was used for chemical analysis and photographs of the obtained crystals (Fig. 4 ▸). Determined mass percentage (+/-3%), calculated mass percentage: K (10.7, 11.4) Fe (12.4, 16.2), Co (13.4, 17.1), P (20.2, 18.0), O (43.3, 37.3)

## 5. Refinement

Crystal data, data collection and structure refinement details are summarized in Table 2 ▸. During the refinement, several models were tested, with the best result for a model with occupational disorder of the two *TM* sites. Since the Co:Fe ratio determined from EDS measurements is almost 1:1, this ratio was constrained for the refinement of the individual site occupation, also taking into account full occupancy of both *TM* sites. For the *TM*1 site a ratio of Fe:Co = 0.5725:0.4275 was obtained, for the *TM*2 site a ratio of Co:Fe = 0.5725/0.4275. The maximum and minimum remaining electron density are located at 0.69 Å and 0.31 Å, respectively, from O8.

## Supplementary Material

Crystal structure: contains datablock(s) I. DOI: 10.1107/S2056989022006521/wm5647sup1.cif

Structure factors: contains datablock(s) I. DOI: 10.1107/S2056989022006521/wm5647Isup2.hkl

CCDC reference: 2181684

Additional supporting information:  crystallographic information; 3D view; checkCIF report

## Figures and Tables

**Table 1:** CHARDI and BVS analysis for the cations in the title compound *q*_(*i*)_ = formal oxidation number; sof_(*i*)_ = site occupation factor; CN_(*i*)_ = classical coordination number; *Q*_(*i*)_ = calculated charge; *V*_(*i*)_ = calculated valence; ECoN_(*i*)_ = effective coordination number.

Cation	*q*_(*i*)_×sof_(*i*)_	CN_(*i*)_	ECoN_(*i*)_	*V*_(*i*)_	*Q*_(*i*)_	*q*_(*i*)_/*Q*_(*i*)_
(Fe/Co)1	2.57	4	4.00	2.48	2.57	1.00
(Fe/Co)2	2.43	4	3.99	2.27	2.43	1.00
K1	1.00	9	8.71	0.94	0.99	1.00
P1	5.00	4	4.00	5.14	5.00	1.00
P2	5.00	4	3.99	5.15	5.01	1.01

**Table 2:** Experimental details

Crystal data	
Chemical formula	KCoFe(PO_4_)_2_
*M*_r_	343.82
Crystal system, space group	Monoclinic, *C*2/*c*
Temperature (K)	296
*a*, *b*, *c* (Å)	13.5860 (6), 13.2320 (6), 8.7316 (4)
β (°)	100.335 (2)
*V* (Å^3^)	1544.21 (12)
*Z*	8
Radiation type	Mo *K*α
μ (mm^−1^)	4.99
Crystal size (mm)	0.36 × 0.27 × 0.15

Data collection	
Diffractometer	Bruker D8 VENTURE Super DUO
Absorption correction	Multi-scan (*SADABS;* Krause *et al.*, 2015 ▸)
*T*_min_, *T*_max_	0.391, 0.747
No. of measured, independent and observed [*I* > 2σ(*I*)] reflections	30042, 3574, 2633
*R*_int_	0.068
(sin θ/λ)_max_ (Å^−1^)	0.820

Refinement	
*R*[*F* ^2^ > 2σ(*F* ^2^)], *wR*(*F* ^2^), *S*	0.036, 0.088, 1.04
No. of reflections	3574
No. of parameters	118
Δρ_max_, Δρ_min_ (e Å^−3^)	0.98, −0.91

Computer programs: *APEX3* and *SAINT* (Bruker, 2016 ▸), *SHELXT2014/7* (Sheldrick, 2015*a* ▸), *SHELXL2018/3* (Sheldrick, 2015*b* ▸), *ORTEP-3 for Windows* (Farrugia, 2012 ▸), *DIAMOND* (Brandenburg, 2006 ▸) and *publCIF* (Westrip, 2010 ▸).

## supplementary crystallographic information

### Crystal data

**Table:**

KCoFe(PO_4_)_2_	*F*(000) = 1328
*M**_r_* = 343.82	*D*_x_ = 2.958 Mg m^−^^3^
Monoclinic, *C*2/*c*	Mo *K*α radiation, λ = 0.71073 Å
*a* = 13.5860 (6) Å	Cell parameters from 3574 reflections
*b* = 13.2320 (6) Å	θ = 2.2–35.6°
*c* = 8.7316 (4) Å	µ = 4.99 mm^−^^1^
β = 100.335 (2)°	*T* = 296 K
*V* = 1544.21 (12) Å^3^	Parallelepiped, purple
*Z* = 8	0.36 × 0.27 × 0.15 mm

### Data collection

**Table:**

Bruker D8 VENTURE Super DUO diffractometer	3574 independent reflections
Radiation source: INCOATEC IµS micro-focus source	2633 reflections with *I* > 2σ(*I*)
HELIOS mirror optics monochromator	*R*_int_ = 0.068
Detector resolution: 10.4167 pixels mm^-1^	θ_max_ = 35.6°, θ_min_ = 2.2°
φ and ω scans	*h* = −13→22
Absorption correction: multi-scan (*SADABS;* Krause *et al.*, 2015)	*k* = −21→21
*T*_min_ = 0.391, *T*_max_ = 0.747	*l* = −14→14
30042 measured reflections	

### Refinement

**Table:**

Refinement on *F*^2^	0 restraints
Least-squares matrix: full	Primary atom site location: structure-invariant direct methods
*R*[*F*^2^ > 2σ(*F*^2^)] = 0.036	Secondary atom site location: difference Fourier map
*wR*(*F*^2^) = 0.088	*w* = 1/[σ^2^(*F*_o_^2^) + (0.0363*P*)^2^ + 2.2954*P*] where *P* = (*F*_o_^2^ + 2*F*_c_^2^)/3
*S* = 1.04	(Δ/σ)_max_ = 0.001
3574 reflections	Δρ_max_ = 0.98 e Å^−^^3^
118 parameters	Δρ_min_ = −0.91 e Å^−^^3^

### Special details

**Table:**

Geometry. All esds (except the esd in the dihedral angle between two l.s. planes) are estimated using the full covariance matrix. The cell esds are taken into account individually in the estimation of esds in distances, angles and torsion angles; correlations between esds in cell parameters are only used when they are defined by crystal symmetry. An approximate (isotropic) treatment of cell esds is used for estimating esds involving l.s. planes.

### Fractional atomic coordinates and isotropic or equivalent isotropic displacement parameters (Å^2^)

**Table:**

	*x*	*y*	*z*	*U*_iso_*/*U*_eq_	Occ. (<1)
Fe1	0.37263 (3)	0.06558 (3)	0.61452 (4)	0.01728 (8)	0.5725
Co1	0.37263 (3)	0.06558 (3)	0.61452 (4)	0.01728 (8)	0.4275
Co2	0.07555 (3)	0.11785 (3)	0.04344 (4)	0.01854 (8)	0.5725
Fe2	0.07555 (3)	0.11785 (3)	0.04344 (4)	0.01854 (8)	0.4275
P1	0.42702 (5)	0.14198 (5)	−0.01872 (7)	0.01656 (12)	
P2	0.14880 (5)	0.06783 (5)	0.41434 (7)	0.01769 (12)	
K1	0.31255 (6)	0.25345 (6)	0.27514 (8)	0.03896 (17)	
O1	0.39529 (17)	0.07417 (15)	0.1059 (2)	0.0288 (4)	
O2	0.54020 (16)	0.13970 (16)	−0.0087 (2)	0.0313 (4)	
O3	0.39339 (19)	0.24769 (14)	0.0152 (3)	0.0338 (5)	
O4	0.37488 (19)	0.1089 (2)	−0.1801 (2)	0.0411 (6)	
O5	0.14870 (17)	−0.04718 (15)	0.4068 (2)	0.0312 (4)	
O6	0.1372 (2)	0.11411 (18)	0.2542 (2)	0.0431 (6)	
O7	0.24615 (14)	0.11037 (15)	0.5076 (2)	0.0262 (4)	
O8	0.06510 (17)	0.1025 (2)	0.4989 (3)	0.0475 (7)	

### Atomic displacement parameters (Å^2^)

**Table:**

	*U*^11^	*U*^22^	*U*^33^	*U*^12^	*U*^13^	*U*^23^
Fe1	0.01435 (16)	0.02100 (16)	0.01659 (14)	0.00191 (12)	0.00307 (11)	−0.00249 (11)
Co1	0.01435 (16)	0.02100 (16)	0.01659 (14)	0.00191 (12)	0.00307 (11)	−0.00249 (11)
Co2	0.01525 (16)	0.02218 (16)	0.01902 (15)	0.00088 (12)	0.00533 (11)	0.00266 (11)
Fe2	0.01525 (16)	0.02218 (16)	0.01902 (15)	0.00088 (12)	0.00533 (11)	0.00266 (11)
P1	0.0185 (3)	0.0157 (2)	0.0169 (2)	0.0005 (2)	0.0071 (2)	−0.00144 (19)
P2	0.0128 (3)	0.0229 (3)	0.0171 (2)	−0.0018 (2)	0.0021 (2)	0.0034 (2)
K1	0.0399 (4)	0.0476 (4)	0.0342 (3)	−0.0028 (3)	0.0197 (3)	−0.0083 (3)
O1	0.0370 (12)	0.0246 (9)	0.0273 (9)	−0.0062 (8)	0.0130 (8)	0.0033 (7)
O2	0.0196 (10)	0.0354 (11)	0.0416 (11)	0.0015 (8)	0.0129 (8)	−0.0039 (9)
O3	0.0478 (14)	0.0212 (9)	0.0388 (11)	0.0109 (8)	0.0254 (10)	0.0033 (8)
O4	0.0407 (14)	0.0625 (16)	0.0201 (9)	−0.0067 (11)	0.0057 (9)	−0.0133 (9)
O5	0.0352 (12)	0.0231 (9)	0.0373 (11)	−0.0083 (8)	0.0116 (9)	0.0011 (8)
O6	0.0560 (16)	0.0449 (13)	0.0247 (10)	−0.0090 (11)	−0.0027 (10)	0.0135 (9)
O7	0.0160 (9)	0.0289 (10)	0.0314 (10)	−0.0003 (7)	−0.0019 (7)	−0.0038 (7)
O8	0.0173 (11)	0.0795 (19)	0.0482 (14)	0.0017 (11)	0.0124 (10)	−0.0163 (13)

### Geometric parameters (Å, º)

**Table:**

Fe1/Co1—O4^i^	1.877 (2)	P2—O5	1.523 (2)
Fe1/Co1—O1^ii^	1.8783 (19)	P2—O7	1.5308 (19)
Fe1/Co1—O7	1.8972 (19)	P2—O8	1.533 (2)
Fe1/Co1—O2^iii^	1.900 (2)	K1—O3	2.694 (2)
Co2/Fe2—O6	1.881 (2)	K1—O7^vii^	2.832 (2)
Co2/Fe2—O8^iv^	1.891 (2)	K1—O6	2.991 (3)
Co2/Fe2—O3^v^	1.9191 (19)	K1—O2^iii^	2.994 (2)
Co2/Fe2—O5^vi^	1.927 (2)	K1—O8^vii^	3.019 (3)
P1—O3	1.5172 (19)	K1—O7	3.029 (2)
P1—O4	1.524 (2)	K1—O1	3.110 (2)
P1—O2	1.525 (2)	K1—O4^v^	3.120 (3)
P1—O1	1.5306 (19)	K1—O5^viii^	3.172 (2)
P2—O6	1.509 (2)		
			
O4^i^—Fe1/Co1—O1^ii^	111.34 (10)	O7^vii^—K1—O7	78.19 (6)
O4^i^—Fe1/Co1—O7	103.45 (10)	O6—K1—O7	47.65 (5)
O1^ii^—Fe1/Co1—O7	115.36 (9)	O2^iii^—K1—O7	58.15 (5)
O4^i^—Fe1/Co1—O2^iii^	113.73 (10)	O8^vii^—K1—O7	98.73 (7)
O1^ii^—Fe1/Co1—O2^iii^	111.57 (9)	O3—K1—O1	48.80 (5)
O7—Fe1/Co1—O2^iii^	100.83 (9)	O7^vii^—K1—O1	166.58 (6)
O6—Co2/Fe2—O8^iv^	116.47 (11)	O6—K1—O1	81.51 (7)
O6—Co2/Fe2—O3^v^	101.81 (11)	O2^iii^—K1—O1	71.69 (6)
O8^iv^—Co2/Fe2—O3^v^	108.09 (12)	O8^vii^—K1—O1	126.06 (7)
O6—Co2/Fe2—O5^vi^	113.85 (11)	O7—K1—O1	91.04 (5)
O8^iv^—Co2/Fe2—O5^vi^	116.25 (11)	O3—K1—O4^v^	103.24 (7)
O3^v^—Co2/Fe2—O5^vi^	97.02 (9)	O7^vii^—K1—O4^v^	59.48 (5)
O3—P1—O4	109.84 (14)	O6—K1—O4^v^	74.98 (7)
O3—P1—O2	110.05 (13)	O2^iii^—K1—O4^v^	152.69 (6)
O4—P1—O2	110.14 (13)	O8^vii^—K1—O4^v^	97.61 (7)
O3—P1—O1	105.59 (12)	O7—K1—O4^v^	102.49 (6)
O4—P1—O1	110.20 (13)	O1—K1—O4^v^	131.79 (6)
O2—P1—O1	110.93 (12)	O3—K1—O5^viii^	58.15 (5)
O6—P2—O5	111.47 (13)	O7^vii^—K1—O5^viii^	84.15 (6)
O6—P2—O7	106.28 (13)	O6—K1—O5^viii^	133.14 (6)
O5—P2—O7	112.60 (12)	O2^iii^—K1—O5^viii^	127.49 (6)
O6—P2—O8	111.23 (16)	O8^vii^—K1—O5^viii^	71.38 (7)
O5—P2—O8	108.96 (14)	O7—K1—O5^viii^	162.10 (6)
O7—P2—O8	106.18 (13)	O1—K1—O5^viii^	106.85 (5)
O3—K1—O7^vii^	142.04 (6)	O4^v^—K1—O5^viii^	65.29 (6)
O3—K1—O6	111.80 (7)	O3—K1—O3^v^	77.20 (8)
O7^vii^—K1—O6	96.69 (7)	O7^vii^—K1—O3^v^	102.00 (5)
O3—K1—O2^iii^	103.65 (7)	O6—K1—O3^v^	54.25 (5)
O7^vii^—K1—O2^iii^	95.61 (6)	O2^iii^—K1—O3^v^	149.32 (6)
O6—K1—O2^iii^	99.16 (6)	O8^vii^—K1—O3^v^	140.19 (7)
O3—K1—O8^vii^	107.98 (8)	O7—K1—O3^v^	101.04 (5)
O7^vii^—K1—O8^vii^	49.37 (6)	O1—K1—O3^v^	87.81 (5)
O6—K1—O8^vii^	140.19 (7)	O4^v^—K1—O3^v^	44.42 (5)
O2^iii^—K1—O8^vii^	69.51 (7)	O5^viii^—K1—O3^v^	79.62 (6)
O3—K1—O7	139.67 (6)		

Symmetry codes: (i) *x*, *y*, *z*+1; (ii) *x*, −*y*, *z*+1/2; (iii) −*x*+1, *y*, −*z*+1/2; (iv) −*x*, *y*, −*z*+1/2; (v) −*x*+1/2, −*y*+1/2, −*z*; (vi) *x*, −*y*, *z*−1/2; (vii) −*x*+1/2, −*y*+1/2, −*z*+1; (viii) −*x*+1/2, *y*+1/2, −*z*+1/2.
